# Feasibility, coverage and cost of oral cholera vaccination conducted by icddr,b using the existing national immunization service delivery mechanism in rural setting Keraniganj, Bangladesh

**DOI:** 10.1080/21645515.2018.1528833

**Published:** 2018-11-28

**Authors:** Ashraful Islam Khan, Iqbal Ansary Khan, Shah Alam Siddique, Anisur Rahman, Md. Taufiqul Islam, Md Amirul Islam Bhuiya, Nirod Chandra Saha, Prasanta Kumar Biswas, Amit Saha, Fahima Chowdhury, Firdausi Qadri

**Affiliations:** a Infectious Diseases Division, International Centre for Diarrhoeal Disease Research, Bangladesh (icddr,b), Dhaka, Bangladesh; b Medical Social Science, Institute of Epidemiology, Disease Control and Research (IEDCR), Dhaka, Bangladesh

**Keywords:** Cholera, oral cholera vaccine (OCV), rural Bangladesh, feasibility, coverage, cost

## Abstract

**Background**: Cholera is a considerable health burden in developing country settings including Bangladesh. The oral cholera vaccine (OCV) is a preventative tool to control the disease. The objective of this study was to describe whether the International Centre for Diarrheal Disease Research, Bangladesh (icddr,b), could provide the OCV to rural communities using existing government infrastructure.

**Methods**: The study was conducted in rural sub-district Keraniganj, 20 km from the capital city Dhaka. All listed participants one year and above in age (excluding pregnant women) were offered two doses of OCV at a 14 day interval. Existing government facilities were used to deliver and also maintain the cold chain required for the vaccine. All events related to vaccination were recorded at the 17 vaccination sites to evaluate the coverage and feasibility of OCV program.

**Results**: A total of 29,029 individuals received the 1st dose (90% of target) and 26,611 individuals received the 2nd dose (83% of target and 92% of 1st dose individuals) of OCV. The highest vaccination coverage was in younger children (1–9 years) and the lowest was amongst 18–29-year age group. Somewhat better coverage was seen amongst the female participants than males (92% vs. 88% for the 1st dose and 93% vs. 90% for the 2nd dose). The cost of vaccine cost was calculated as US$1.00 per dose plus freight, insurance, and transportation and the total vaccine delivery cost was US$70,957.

**Conclusion**: This was a project undertaken using existing public health program resources to collect empirical evidence on the use of a mass OCV campaign in the rural setting. Mass vaccination with the OCV is feasible in the rural setting using existing governmental vaccine delivery systems in Bangladesh.

## Introduction

Cholera, in spite of persistent global efforts, is still the major cause of morbidity and mortality worldwide. Cholera is endemic in Africa and Asia including in Bangladesh and has recently spread to the Americas. An estimated 1.3 billion people worldwide are at risk of cholera, with India and Bangladesh constituting the largest share of this population at risk.^1^ An estimated 3 million cases and 95,000 deaths occur in 69 endemic countries each year, particularly where access to water and sanitation infrastructure is inadequate.^1–3^ According to the World Health Organization (WHO), only 5–10% of cholera cases are actually reported, and it is likely that their data on cholera rates are a gross underestimation of the real disease burden.^4^

Due to an absence of surveillance and diagnostic facilities, the exact burden of cholera in Bangladesh could not be evaluated. Recently, the International Centre for Diarrheal Disease Research, Bangladesh (icddr,b), in collaboration with the Government of Bangladesh, established a nationwide surveillance program for cholera at 22 sites including at the district and sub-district levels, which cover all the divisions of Bangladesh. The disease control room of the Directorate General of Health Services (DGHS) maintains a diarrhea surveillance cell on reported cases from different health facilities in urban and rural areas. Based on this, the Institute of Epidemiology, Disease Control Research (IEDCR) estimates that there are around 450,000 cases of cholera each year in Bangladesh.^5^ Other studies have shown that cholera is widely distributed and present in rural areas situated in different geographical areas of Bangladesh, with each area having a different pattern of cholera outbreaks.^6^

Oral rehydration therapy (ORT) is a key component of the management of diarrhea, with intravenous infusions (IV) sometimes needed even though this does not reduce the incidence or eliminate the pathogen, *Vibrio cholerae*. Moreover, frequent antimicrobial use is rapidly diminishing their effectiveness due resistance against enteric pathogens. A few large vaccine trials conducted in Bangladesh revealed that the oral cholera vaccine (OCV) could substantially prevent cholera.^7–9^ Therefore, OCV use along with other control measures has become an important public health tool for the prevention and control of cholera.^10,11^ Moreover, periodic OCV campaigns every three or five years could significantly reduce the global burden of cholera.^12–14^

A large feasibility study with Shanchol^TM^ OCV was conducted in Mirpur in 2011 to test vaccine delivery strategies in urban high-risk areas (13). To reduce the burden of cholera and associated morbidity and mortality in rural areas, we proposed to test the feasibility of implementing the OCV in the rural setting through appropriate and feasible delivery mechanisms using the existing EPI service delivery system. The objective of this study was to analyze whether the OCV can be provided to rural communities at risk of cholera through the existing government system and whether such a program is feasible for government implementation in similar settings.

## Results

**Figure 1.**
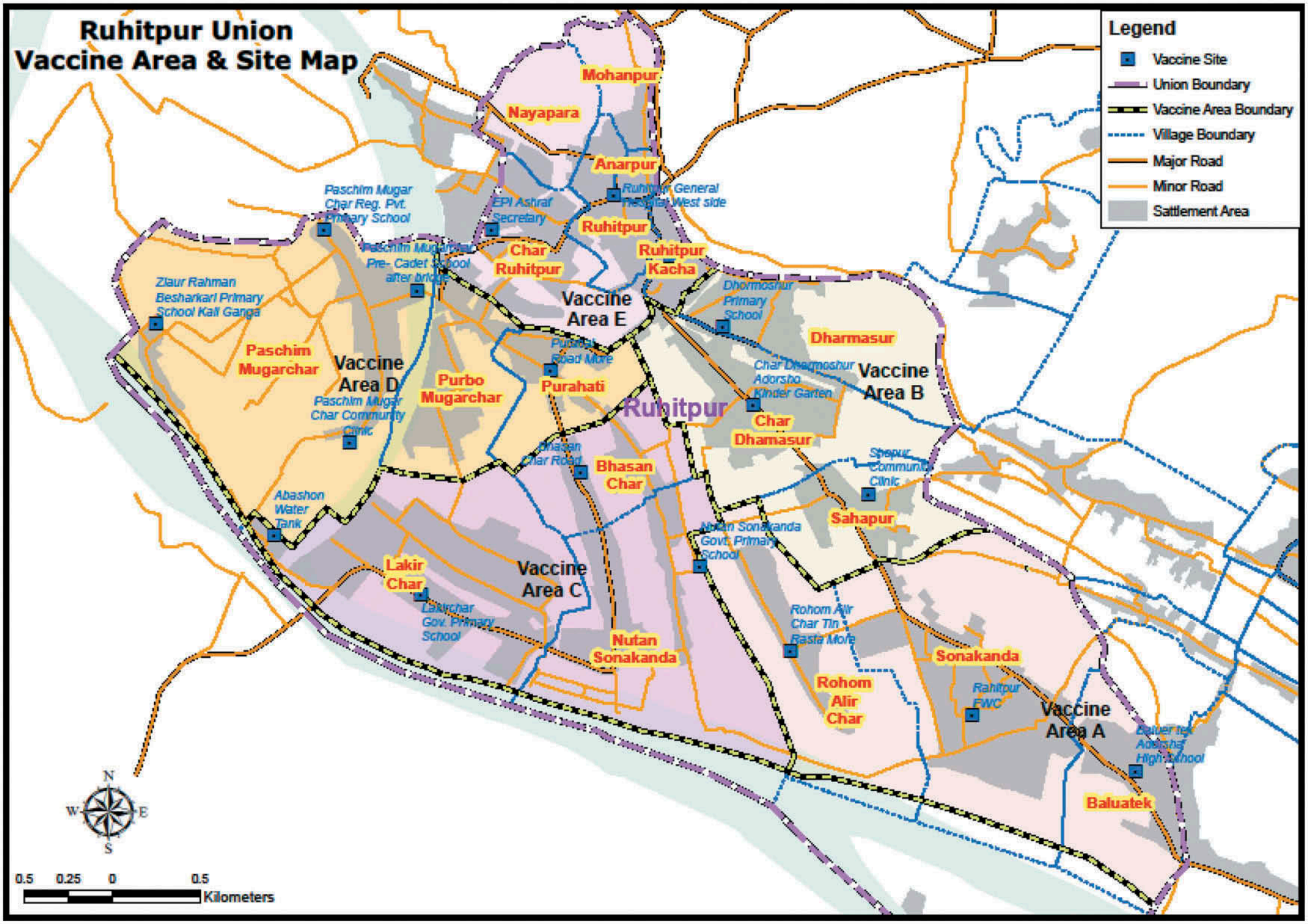
Vaccination sites in the study area.

**Figure 2.**
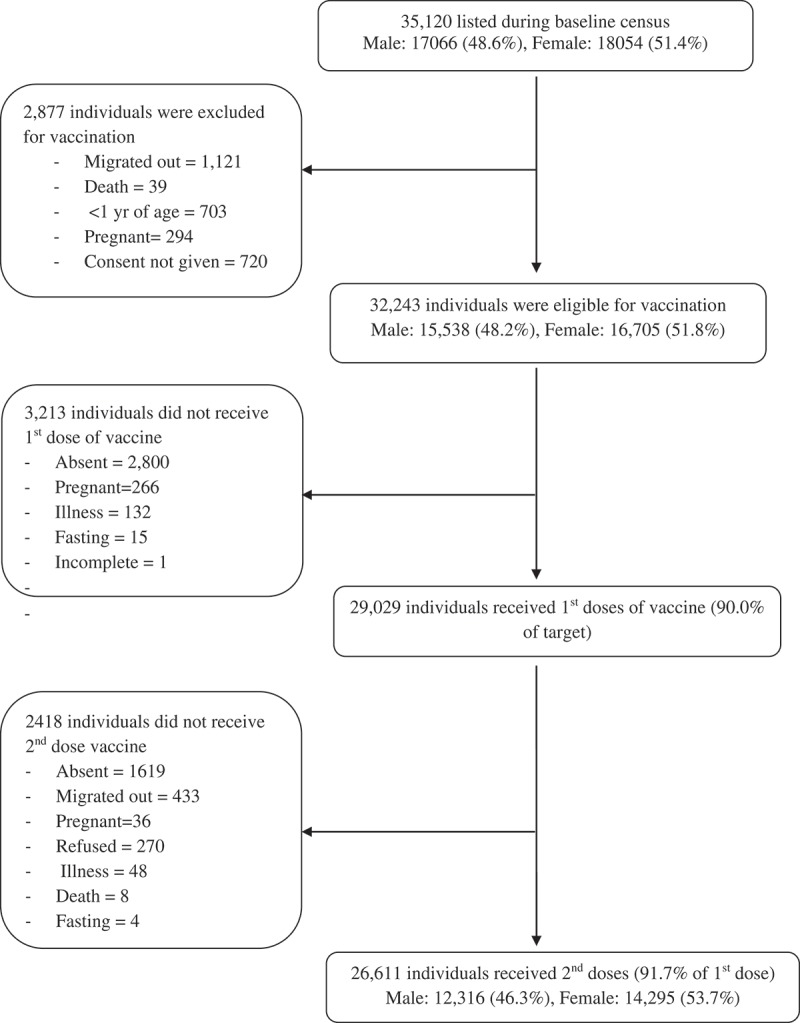
CONSORT chart showing recorded complete vaccine doses among the targeted high-risk cholera population.

35,120 subjects were listed in the baseline survey as a high-risk population for vaccination. 2,877 of these were not targeted for vaccination due to being <1 year of age, unwilling to give consent, pregnant, or having migrated out or died. During vaccination, 29,029 individuals received a complete 1st dose (90% of target) of vaccine and 26,611 individuals received the 2nd dose (92% of 1st dose) of vaccine (Figure 1 and Figure 2).

The study population characteristics and a comparison of vaccine recipients and non-recipients are shown in Table 1. Children, females, and people with their own house participated significantly more in the vaccination program. The major causes of not participating in the vaccination were being absent on the day of vaccination, pregnancy, illness, and fasting during the period of the campaign. About 41% of study participants were 17 years of age or younger and about 14% were 50 years or older. Forty-eight percent of participants were male and 79% had their own household. About 81% of participant house construction material was tin, and tube well was the main source of drinking water (67%). Only 0.4% reported diarrhea in the 7 days prior to the baseline visit in that season.

**Table 1.** Background characteristics of population eligible for vaccination.

Characteristics	Total n (%)	Vaccinated n (%)	Non-vaccinated n (%)	p-value	COR (95% CI)
**Age in years**					
*1–9*	6882 (21.3)	6536 (22.5)	346 (10.8)		Ref
*10–17*	6225 (19.3)	5789 (19.9)	436 (13.6)	0.00	0.71 (0.63, 0.81)
*18–29*	7007 (21.7)	5931 (20.4)	1076 (33.5)	0.00	0.28 (0.25, 0.31)
*30–39*	4378 (13.6)	3875 (13.3)	503 (15.7)	0.00	0.37 (0.33, 0.41)
*40–49*	3364 (10.4)	2992 (10.3)	372 (11.6)	0.00	0.39 (0.34, 0.44)
*50–59*	2049 (6.4)	1845 (6.4)	204 (6.3)	0.00	0.42 (0.37, 0.49)
*60+*	2338 (7.3)	2061 (7.1)	277 (8.6)	0.00	0.39 (0.34, 0.44)
**Sex**					
*Male*	15,538 (48.2)	13,612 (46.9)	1926 (59.9)		Ref
*Female*	16,705 (51.8)	15,417 (53.1)	1288 (40.1)	0.00	1.55 (1.46, 1.64)
**Education (n = 29,301)**					
*Primary*	11,315 (38.6)	10,239 (39)	1076 (35.3)		Ref
*Secondary*	7906 (27)	7022 (26.7)	884 (29)	0.00	0.83 (0.76, 0.92)
*Higher secondary or higher*	910 (3.1)	780 (3.0)	130 (4.3)	0.00	0.63 (0.52, 0.77)
*No formal education*	2736 (9.3)	2469 (9.4)	267 (8.8)	0.69	0.97 (0.84, 1.12)
*No education*	6434 (22)	5741 (21.9)	693 (22.7)	0.01	0.87 (0.79, 0.96)
**Diarrhea within 7 days**					
*Yes*	127 (0.4)	115 (0.4)	12 (0.4)		Ref
*No*	32,116 (99.6)	28,914 (99.6)	3202 (99.6)	0.85	0.94 (0.52, 1.71)
**Family size per room (Grouped)**					
*< 2*	1536 (19)	1228 (19.3)	185 (17.5)		Ref
*2–4*	4168 (51.5)	3262 (51.3)	548 (51.9)	0.41	0.96 (0.86, 1.06)
*> 4*	2383 (29.5)	1864 (29.3)	322 (30.5)	0.12	1.09 (0.98, 1.22)
**Household type**					
*Own*	6371 (78.8)	5117 (80.5)	728 (69.0)		Ref
*Rented*	1548 (19.1)	1115 (17.5)	299 (28.3)	0.00	0.61 (0.56, 0.67)
*Others*	168 (2.1)	122 (1.9)	28 (2.7)	0.03	0.77 (0.6, 0.98)
**Household’s wall materials**					
*Tin*	6541 (80.9)	5176 (81.5)	846 (80.2)		Ref
*Bricks/Cement*	1392 (17.2)	1069 (16.8)	179 (17.0)	0.42	0.96 (0.87, 1.06)
*Others*	154 (1.9)	109 (1.7)	30 (2.8)	0.13	0.83 (0.64, 1.06)
**Source of drinking water**					
*Own Tap*	264 (3.3)	202 (3.2)	36 (3.4)		Ref
*Own hand pump*	5383 (66.6)	4328 (68.1)	621 (58.9)	0.68	1.04 (0.85, 1.28)
*Communal hand pump*	2390 (29.6)	1788 (28.1)	390 (37.0)	0.05	0.81 (0.65, 1.00)
*Others*	50 (0.6)	36 (0.6)	8 (0.8)	0.69	0.9 (0.54, 1.49)

Details of the vaccine delivery plan including 1st and 2nd dose, vaccination team, and logistics are described in **Table S1**, and vaccine coverage is presented in **Table S2**. The overall full dose vaccine coverage was 83%; however, the 2nd dose coverage among 1st dose recipients was about 92%. The major causes for not receiving the 2nd dose were migrating out from the area, being absent, pregnant, physically ill, dead, or fasting during the vaccination period. Vaccine coverage was inversely related to age, i.e., vaccine coverage decreased with increasing age. The highest coverage of vaccination was among younger children (1–9 years) and lowest was for those aged 18–29 years. Female participants had higher coverage than male participants (92% vs. 88% for the 1st dose and 93% vs. 90% for the 2nd dose).

Adverse events following immunization (AEFI) were monitored for 14 days after each dose. In total, nine AEFIs (0.031%) were reported at the vaccination sites or in the two participating hospitals (Keraniganj Health Complex and icddr,b Dhaka Hospital). Among them, five were reported after the first dose and the other four were reported after the second dose. Acute watery diarrhea with or without vomiting, abdominal pain, and fever were reported events during the AEFI monitoring period (Table 2). Among the AEFI cases only two were presented with some sign of dehydration and the rest were no signs of dehydration.

**Table 2.** Adverse events following immunization.

Sign/symptoms	No. of patients	Dose 1	Dose 2	Average duration (in hours)
Acute watery diarrhea with vomiting	2	1	1	05 (02–08)
Acute watery diarrhea with abdominal pain	2	1	1	11 (06–16)
Acute watery diarrhea with fever, vomiting	1	1	0	14 (14)
Acute watery diarrhea with nausea, abdominal discomfort	1	0	1	04 (04)
Acute watery diarrhea with vomiting, abdominal pain and fever	1	1	0	17 (16–18)
Only abdominal pain	2	1	1	04 (04)
**Total**	**9**	**5**	**4**	

The as-received vaccine cost was calculated at US$1.00 per dose for research purposes (although its commercial price was US$1.85) plus freight, insurance, and transportation costs. Costs of wasted vaccines were also included. Cold chain-related logistics, training venues, etc. were received free of charge so their cost was not included. The total expenditure for this vaccination project was US$70,957, including US$59,222 (84%) for vaccine purchase (Table 3). Excluding vaccine purchase, the expenditure for delivery of 26,611 complete doses and 2,418 single doses (55,640 doses) given within the existing EPI infrastructure was $11,735, approximately US$0.21/dose (Table 3).

**Table 3.** Cost of vaccine delivery.

Cost category	Cost US$ (Tk. 77.7 = 1 US$)	Percentage of total cost
Vaccine: Used 55,940 vaccine vials (including freight, insurance)	$59,222.36	83.46
Logistics (furniture, decorator, office cost)	620.05	0.87
Stationary	198.76	0.28
Cost for transportation of logistics during vaccination	1,110.04	1.56
Awareness building cost	45.05	0.06
Vaccination card	362.93	0.51
Master listing and session report, consent and other recording forms	655.89	0.92
Training costs (including manuals, guides)	373.23	0.53
Vaccination related staff salaries (including shared salaries)	5,399.36	7.61
Conveyance cost	470.39	0.66
Food and refreshment for the workers during vaccination	2,207.54	3.11
Waste disposal cost (including incineration)	291.15	0.41
Total	70,956.75	100

## Discussion

This campaign represents the first ever OCV campaign conducted in a rural setting in Bangladesh using existing government facilities to test the adopted strategies. Other vaccination campaigns (e.g., for polio) have been conducted in the same area through government settings. However, in those campaigns, the target age group (0–5 years) was different and, in terms of outreach, community residents were well aware of polio and other EPI vaccination programs and the provider also trained on those campaigns. In contrast, the cholera vaccination campaign required awareness raising since it was a new vaccine. Vaccination coverage for the 1st dose was 85% among the target population and 92% of the 1st dose recipients received the 2nd dose, leaving only 8% dropouts. Most participants were 1–39 years of age. Overall, there was better participation by females for all age groups except 1–9 years, where male participation was higher.

This study reveals that vaccine delivery with high coverage is feasible in the rural setting using existing government facilities. As reported in other studies, the main expenditure is for vaccine purchase^15,16^ Attention is needed to lower this cost for widespread use of this vaccine, and the vaccine cost will be further reduced when locally-produced vaccines eventually become available. Our findings suggest that OCV use in the rural setting in Bangladesh is feasible and targets almost the entire community with high coverage and low cost. However, it also requires intense micro-planning, additional human resources, and cold chain capacities.

Only interpersonal communication and local mosque announcements were used to encourage participants to receive the vaccine. Appropriate communication materials, e.g., microphones, media, posters, and leaflets may help to build awareness and confidence in the program and to mobilize people to participate, thereby further increasing coverage. Such strategies could not be adopted in this study to avoid contamination of the study sample by participation of the population living in adjacent unions. This lack of communication through mass media is likely to have had a significant impact on the number of dropouts and population left out from vaccination. This was a new intervention and an unknown vaccine for the community; regardless, the coverage is a slightly higher than other OCV studies conducted in Bangladesh.^9,12^

In terms of cold chain infrastructure, Shanchol requires more space for storage and transportation as it is a single-dose vial. Conducting mass vaccination campaigns for entire communities poses considerable challenges for the public health infrastructure, particularly with regards to human resources and cold chain capacity in the rural setting. However, it has been shown that vaccine safety and immunogenicity are not altered when the vaccine is kept at ambient temperatures outside the cold chain.^17^ Another large campaign was conducted in Bangladesh in the forcibly displaced Myanmar nationals (FDMN), where vaccine was delivered at ambient temperatures on the day of vaccination.^18^ These experiences are highly relevant to policy makers for further planning.

Two campaigns using Shanchol in Bangladesh and India reported delivery costs per dose of $0.76 and $0.49, respectively, which was higher than the cost of this campaign.^12,19^ The overall cost of vaccine delivery to the high-risk rural area was kept low by using the existing cold chain system, trained and experienced governmental human resources, and excellent community participation. Furthermore, coverage will further improve when this vaccination program is adopted by the well-trusted EPI program of the Government of Bangladesh. Coverage could be further enhanced by proper media communication, facility-based extended service hours, and institutional vaccine delivery systems. Moreover, there were many costs which are not really essential to successful vaccination under the current environment. The careful census, the care recording of vaccination, the excessively careful cold chain, the exclusion of pregnant women is no longer recommended during routine vaccination campaigns. Thus, many of the costs could be reduced further.

## Limitations

This study has several limitations. We did not assess the effect of the behavioral change intervention. This component was not included, as the primary purpose of this project was to assess routine public health implementation of the vaccine. Moreover, the behavioral change intervention is not routine in public health programs in Bangladesh. A second limitation is that the capital costs such as buildings, cold chain rooms, and equipment were not included in cost calculations. Moreover, we analyzed relatively old data (from a few years ago), so the data may not be fully representative of the current period.

## Conclusions

Mass vaccination with OCV targeting high-risk populations in rural setting is feasible in terms of coverage and cost by using existing government vaccine delivery systems. The high coverage reveals excellent community participation and acceptance of the OCV. Coverage is likely to improve and delivery costs decrease when vaccination is carried out as part of regular national immunization programs through government-led mechanisms with proper media communication. This campaign was conducted several years ago, and there has since been progress in terms of experience in delivering OCV. Regardless, this study provides important data on how to deliver the OCV in the rural setting using available EPI facilities in Bangladesh and other endemic countries.

## Materials and methods

### Study sites and population

The Keraniganj upazila of Dhaka district was purposively selected as the study area due to its close proximity to Dhaka and also due to high incidence rates of diarrhea and evidence of culture-confirmed cholera. Based on the Keraniganj diarrheal disease report to the Directorate General of Health Services (DGHS) and cholera prevalence rates in icddr,b surveillance data, the Ruhitpur union (population approximately thirty thousand) was selected as the study area for vaccination.

The study was initiated on 30th July 2012 to observe the feasibility of intervention with the OCV in Ruhitpur union, Keraniganj upazila. The censused population of that union was targeted for vaccination with two doses of OCV at a minimum interval of 14 days between doses. All listed participants above one year excluding pregnant females were offered two doses of vaccine.

### Vaccine

The oral cholera vaccines (OCV) Shanchol^TM^ used in this study were leftover vaccines from the Introduction of Cholera Vaccine in Bangladesh (ICVB, icddr,b protocol #10,061) study conducted in urban Mirpur of Dhaka.^8^ About sixty-five thousand vaccine doses were available after completion of the ICVB vaccination program. Each dose of the liquid bivalent Shanchol^TM^ vaccine contains inactivated whole-cell heat-killed and formalin-killed *Vibrio cholerae* O1 and O139 and described elsewhere.^8,9^ The vaccine has no detectable levels of cholera toxin.

### Census activities

A census was conducted prior to vaccine delivery to identify the eligible target population. For this purpose, and with the help of satellite images (from Google Maps), all villages were digitized and initial village maps were prepared for update by ground truthing. In the subsequent census update, global positioning system (GPS) data for each household in the two unions were gathered using GPS devices to document their actual locations. After proper training, baseline census data collection was initiated on August 3, 2012 and was completed by September 30, 2012. The baseline census using a hard copy census questionnaire identified 35,120 participants from 8,088 households in the Ruhitpur Union. Based on the gathered information, a Rural Oral Cholera Vaccine (ROCV) family card was prepared for each household and delivered just prior to vaccination. Each household received the card with a unique household number and address with all family members’ names, unique individual bar-coded IDs, and basic demographic information. The cards were used for the identification of eligible participants during vaccine delivery.

### Assembling logistics

The vaccines were stored in the cold room of the EPI headquarters in Mohakhali, Dhaka, approximately 20 km away from Ruhitpur Union of the Keraniganj Upazilla in the Dhaka District. For vaccine transportation, twelve cold boxes were requisitioned from the reserve of the EPI and Keraniganj Upazila Heath Complex (UHC), government health facilities at the sub-district levels. Twelve vaccine carriers were also collected from the Keraniganj UHC for vaccine delivery. Required frozen icepacks for the cold boxes were supplied from the EPI freezer room at the time of vaccine transportation. The fast freezer at the Keraniganj UHC was also used for additional frozen ice packs. Keraniganj UHC had three ice-lined refrigerators (ILR); routine EPI vaccines were rearranged in two ILRs, and one ILR was allocated to OCV reservation at the UHC. Vaccines, all other logistics, and field worker transportation were conducted by three pick-up vans throughout the vaccination period. Low cost plastic foldable tables and chairs for one site area (ten tables and fifty chairs) were procured and used for all sites in a phase-wise manner. Further, a few banners and adequate waste collection bags were also procured.

### Recruitment and training of fieldworkers

The existing local health assistants (HAs) were asked to select a required number of local volunteers from each EPI routine outreach site. After proper training, the selected volunteers were involved in household listing and collection of the basic demographic information. They were also engaged to build awareness about the vaccination program against cholera at that time. This was conducted three months prior to vaccination. After completion of baseline data collection, they were trained on advocacy, mobilization of people for vaccination, vaccination strategy, vaccination and session management, record keeping, reporting, verbal consent process for ingesting the vaccine, and distribution of family cards.

### Communication strategy and social mobilization

Communication strategies included inter-personal communication (IPC) by the field workers and focal advocacy meetings. Upazila Health and Family Planning Officers (UHFPOs), people involved in the EPI, and community leaders were involved in advocacy meetings. Prior to vaccination, trained workers and volunteers visited each target household to distribute cards and present the messages related to cholera, OCV, and vaccination activities. Communities were reminded by the volunteers about their vaccination date and time and to bring the family card to the vaccination site. Banners at vaccination sites were used to create awareness and encourage participation. Mosque megaphones were used to let the participants know about the OCV program and to invite them to participate in the program at scheduled dates and times.

### Vaccine delivery strategy and implementation

Vaccines were stored in the EPI cold room at 2–8°C. Existing EPI cold chain facilities at the Keraniganj Upazila Health Complex were used to deliver the vaccine in the selected union. Trained community healthcare providers, health assistants, family welfare assistants (FWAs), and locally recruited volunteers worked as vaccinators and volunteers. Adults and older children received the vaccine by themselves but younger children received the vaccine(s) with the help of a vaccinator.

The vaccination program was carried out between October 6 and November 22, 2012, with an initial launch ceremony with local and national dignitaries on October 4 and a few vaccinations in a community clinic in Ruhitpur. The intervention population was divided into five vaccination areas, A, B, C, D and E (Figure 1), each containing around 6,000 in the target population to be vaccinated in three days. There were three temporary vaccination outreach sites for areas A, B, and E, whereas areas C and D each had four sites. The vaccination program was carried out for three days in each area. In each vaccination area, 45 members were involved, 20 as vaccinators, 10 as record keepers, 5 as mobilizer/AEFI monitors, 5 as gatekeepers, 4 as supervisors, and 1 coordinator. Sites were established in community clinics, Health and Family Welfare Centers (FWCs), schools, marketplaces, and existing EPI sites. On the 2nd and 3rd day, a few sub-centers were established along with the main center to ensure coverage. In each area, on the third day, along with the temporary fixed site vaccination, mop-up activities were carried out by visiting households to cover those left out. After completion of a vaccination area in three days, the vaccination team moved to the next area. All the eligibility criteria (age over one year, pregnancy, health status for any acute illness like diarrhea, fever, etc.) were checked by verbal screening prior to vaccination for each participant.

### Cold chain, session management, and record keeping

On the day of vaccination, the teams set up the sites, informed and mobilized the target population, and delivered the vaccines. Vaccination histories were recorded in the master list and session report form for each site. Date of vaccination was also captured on the vaccination card. These session report forms contained the eligible target list in each catchment area, scheduled vaccination dates, and information regarding the individual’s eligibility. These master list and session report forms helped to track down those left out or dropped out from each site.

On the first vaccination day, twelve cold boxes with vaccines (each with 750 vials) and conditioned icepacks were shifted from the EPI store to Keraniganj UHC in the early morning at around 6.00 am. Out these, seven boxes were moved to the ILR at 2–8°C at the UHC store as reserves for future use. The other five boxes were supplied to the main vaccination fixed outreach site scheduled on that day along with 10 vaccine carriers. From the vaccination site, required logistics were distributed to the sub-sites. During vaccination sessions, vaccines were occasionally moved from the cold box to the vaccine carriers with conditioned icepacks and administered to participants.

At the end of the session, empty cold boxes were returned along with waste bags to the UHC. The remaining vaccines were also kept in the ILR of UHC. On the second vaccination day, 5 cold boxes containing around 3,500 doses were sent to the site from the UHC store. On the third day, the required vaccines were sent to the sites from UHC and, according to left out status, one or two vaccination teams (one vaccinator and one tally marker in each team) conducted vaccination sessions at the temporary fixed sites and the rest were engaged in mop-up activities with left-outs within the catchment area. Routine immunization activities by the EPI field workers were not disrupted. Cold boxes (along with vaccine carriers) were returned on the third day to the UHC. For the next vaccination area, again the required vaccines were moved from the EPI store and a reserve was always kept at the UHC store.

### Adverse events following immunization (AEFI) management

Adverse events were monitored for 14 days after each dose. At the vaccination sessions, all participants were asked to wait for 30 minutes at the site after taking the vaccine. One member of staff was delegated to monitor any immediate adverse events and to notify the assigned study physician if any occurred. Each event was reported through a pre-set questionnaire. Keraniganj Upazila Health Complex was designated as the AEFI case management center. Participants were also told to report any untoward effects to the AEFI case management center. Causal relationships between detected events and vaccination were assessed by review of case report forms by experienced clinicians and a central AEFI committee.

### Data analysis

The data used for analysis was entered into ‘Visual FoxPro 6ʹ software. The cholera vaccination status was ascertained from the vaccination cards and history. The vaccine coverage for each vaccination round and the proportion of coverage and drop-outs were calculated. The vaccine coverage was also stratified by age and sex. All resources used during vaccination process were recorded and shown under different cost categories. Based on the time spent on vaccination activities, shared salaries were calculated for hospital staff and second-line supervisors. Data analyses were performed in SPSS for Windows, version 17.5 (SPSS Inc., Chicago, IL).

## SUPPLEMENTAL DATA

Supplemental data for this article can be access at tandfonline.com/khvi.
