# Supplementary material for: Feasibility, coverage and cost of oral cholera vaccination conducted by icddr,b using the existing national immunization service delivery mechanism in rural setting Keraniganj, Bangladesh
Source: Hum Vaccin Immunother. 2018 Nov 28;15(6):1302–9. doi: 10.1080/21645515.2018.1528833 (PMC6663147; doi:10.1080/21645515.2018.1528833)
Supplement: Supplemental Material [file khvi-15-06-1528833-s001.zip › KHVI_A_1528833_Table S1.docx]

**Table S1: Vaccination Plan**

| Vaccination area | First Round  (Dose 01)  (17 days) | Dose Interval (from last recipient of the site) | Second Round  (Dose 02)  (17 days) | Population targeted (34 days for two dose) | Strategies | Human resource, vaccine and cold chain requirement |
| --- | --- | --- | --- | --- | --- | --- |
| A | **Day 1 & 2:**  6-7/10/12 | At least 14 days interval | **Day 1 & 2:**  3-4/11/12 | Around 6000 targeted population | **Day 1 & 2:**  Regular cholera vaccination at the temporary fixed sites along with required sub-site/s.  **Day 3**  OCV left out (at first round) and drop out (at second round) coverage through mop-up activities at house hold level along with the temporary fixed site/s. | **HR requirement vaccination days:**  20 vaccinator + 10 tally marker + 5 gate keeper + 5 mobilizer/AEFI monitor + 4 supervisor + 1 coordinator  **Vaccine requirements:**  On the first and second vaccination days, around 3000-3500 vaccine vials each day. On the third day, required vaccines will be sent according to the left out/drop out list.  **Cold chain requirements:**  Total 12 cold boxes, 12 vaccine carriers and 672 small icepacks |
|  | **Day 3:** 8/10/12 |  | **Day 3: 5**/11/12 |  |  |  |
| B | **Day 1 & 2:**  9-10/10/12 | At least 14 days interval | **Day 1 & 2:**  6-7/11/12 | Around 6000 targeted population |  |  |
|  | **Day 3:** 11/10/12 |  | **Day 3: 8**/11/12 |  |  |  |
| C | **Day 1 & 2:**  13-14/10/12 | At least 14 days interval | **Day 1 & 2:**  10-11/11/12 | Around 6000 targeted population |  |  |
|  | **Day 3:** 15/10/12 |  | **Day 3: 12**/11/12 |  |  |  |
| D | **Day 1 & 2:**  16-17/10/12 | At least 14 days interval | **Day 1 & 2:**  13-14/11/12 | Around 6000 targeted population |  |  |
|  | **Day 3:** 18/10/12 |  | **Day 3: 15**/11/12 |  |  |  |
| E | **Day 1 & 2:**  20-21/10/12 | At least 14 days interval | **Day 1 & 2:**  17-18/11/12 | Around 6000 targeted population |  |  |
|  | **Day 3:** 22/10/12 |  | **Day 3: 19**/11/12 |  |  |  |
