# Supplementary material for: Feasibility, coverage and cost of oral cholera vaccination conducted by icddr,b using the existing national immunization service delivery mechanism in rural setting Keraniganj, Bangladesh
Source: Hum Vaccin Immunother. 2018 Nov 28;15(6):1302–9. doi: 10.1080/21645515.2018.1528833 (PMC6663147; doi:10.1080/21645515.2018.1528833)
Supplement: Supplemental Material [file khvi-15-06-1528833-s001.zip › KHVI_A_1528833_Table S2.docx]

| **Table S2. Complete vaccine coverage by age group and sex against total targeted population** | | | | | | | | | |
| --- | --- | --- | --- | --- | --- | --- | --- | --- | --- |
| **Age (years)** | **Targeted population^a^  N (%)** | | | **Dose 1 coverage** | | | **^b^Dose 2 coverage** | | |
|  |  |  |  | **N (%)** | | | **N (%)** | | |
|  | **Male** | **Female** | **Total** | **Male** | **Female** | **Total** | **Male** | **Female** | **Total** |
| 1-9 | 3,503 | 3,379 | 6,882 | 3,326 (95) | 3,210 (95) | 6,536 (95) | 3,149 (95) | 3,004 (94) | 6,153 (94) |
| 10-17 | 3,225 | 3,000 | 6,225 | 2,982 (92) | 2,807 (94) | 5,789 (93) | 2,772 (93) | 2,618 (93) | 5,390 (93) |
| 18-29 | 3,022 | 3,985 | 7,007 | 2,489 (82) | 3,442 (86) | 5,931 (85) | 2,164 (87) | 3,089 (90) | 5,253 (89) |
| 30-39 | 1,845 | 2,533 | 4,378 | 1,499 (81) | 2,376 (94) | 3,875 (89) | 1,256 (84) | 2,209 (93) | 3,465 (89) |
| 40-49 | 1,636 | 1,728 | 3,364 | 1,341 (82) | 1,651 (96) | 2,992 (89) | 1,176 (88) | 1,561 (95) | 2,737 (91) |
| 50-59 | 1,021 | 1,028 | 2,049 | 867 (85) | 978 (95) | 1,845 (90) | 769 (89) | 926 (95) | 1,695 (92) |
| 60+ | 1,286 | 1,052 | 2,338 | 1,108 (86) | 953 (91) | 2,061 (88) | 1,030 (93) | 888 (93) | 1,918 (93) |
| All | 15,538 | 16,705 | 32,243 | 13,612 (88) | 15,417 (92) | 29,029 (90) | 12,316 (90) | 14,295 (93) | 26,611 (92) |
| ^a^ Total targeted high-risk population (excluding <1yr and pregnant woman) | | | | | | | | | |
| ^b^ 2^nd^ dose immunization calculated over 1^st^ dose coverage | | | | | | | | | |
